# Default-mode network activity is retained in the isolated hemisphere of people after hemispherotomy

**DOI:** 10.1093/braincomms/fcag211

**Published:** 2026-06-16

**Authors:** Tobias Bauer, Charlotte Gauvry, Sebastian Markett, Thomas Kreter-Schönleber, Valeri Borger, Hartmut Vatter, Josemir W Sander, Markus Gabriel, Alexander Radbruch, Rainer Surges, Theodor Rüber

**Affiliations:** Department of Neuroradiology, University Hospital Bonn, Bonn 53127, Germany; Department of Epileptology, University Hospital Bonn, Bonn 53127, Germany; German Center for Neurodegenerative Diseases (DZNE), Bonn 53127, Germany; International Center for Philosophy, University of Bonn, Bonn 53113, Germany; Department of Psychology, Humboldt University Berlin, Berlin 10117, Germany; Department of General Psychiatry, Heidelberg University Hospital, Heidelberg 69120, Germany; Department of Neurosurgery, University Hospital Bonn, Bonn 53127, Germany; Department of Neurosurgery, University Hospital Bonn, Bonn 53127, Germany; Department of Clinical & Experimental Epilepsy, UCL Queen Square Institute of Neurology, London WC1N 3BG, UK; Chalfont Centre for Epilepsy, Chalfont St Peter SL9 0RJ, UK; Department of Neurology, West China Hospital, Sichuan University, Chengdu 610041, China; International Center for Philosophy, University of Bonn, Bonn 53113, Germany; Department of Neuroradiology, University Hospital Bonn, Bonn 53127, Germany; German Center for Neurodegenerative Diseases (DZNE), Bonn 53127, Germany; Center for Medical Data Usability and Translation, University of Bonn, Bonn 53113, Germany; Department of Epileptology, University Hospital Bonn, Bonn 53127, Germany; Department of Neuroradiology, University Hospital Bonn, Bonn 53127, Germany; Department of Epileptology, University Hospital Bonn, Bonn 53127, Germany; German Center for Neurodegenerative Diseases (DZNE), Bonn 53127, Germany; Center for Medical Data Usability and Translation, University of Bonn, Bonn 53113, Germany

**Keywords:** epilepsy surgery, functional MRI, consciousness

## Abstract

Hemispherotomy is a surgical intervention for severe drug-resistant epilepsy that fully disconnects the lesional brain hemisphere from the rest of the nervous system. This creates a rare opportunity to explore whether consciousness can be sustained without external input. The default-mode network, linked to introspective mental activity, is considered a necessary, though not sufficient, condition for consciousness. In this prospective case-control study, resting-state functional MRI data from 26 individuals post-hemispherotomy and 24 healthy controls were analysed. Default-mode network activity was assessed using seed-to-voxel correlations based on the peak activity in the precuneus of controls, delineated by group independent component analysis. In connected hemispheres, typical default-mode network patterns were preserved and did not differ significantly from controls. Isolated hemispheres also showed preserved positive default-mode network connectivity, though negative connectivity was significantly reduced compared to controls. In two patients with both pre- and post-surgical scans, the individual default-mode network remained detectable but was divided between the isolated and connected hemispheres. These findings suggest that default-mode network activity persists in isolated hemispheres, meeting a necessary condition for consciousness. However, the presence of default-mode network activity alone does not confirm conscious awareness, encouraging further investigation into the neural correlates of consciousness in isolated hemispheres.

## Teaser

Bauer *et al*. report that default-mode network activity can be detected with resting-state functional MRI in the isolated hemisphere following hemispherotomy, satisfying a necessary condition for consciousness. They further show, in two longitudinal cases, that pre-surgical default-mode network connectivity is preserved after surgery, but becomes divided between hemispheres.

## Graphical Abstract

**Graphical Abstract:**
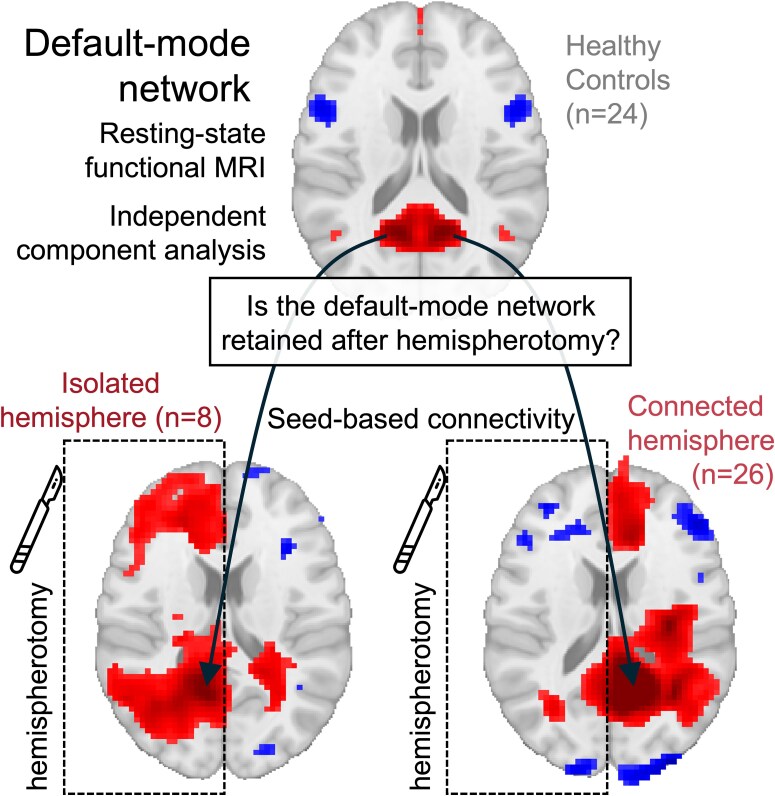

## Introduction

Hemispherotomy (HT) is a neurosurgical procedure that is performed as an ultima-ratio in severe cases of drug-resistant epilepsy with unihemispheric lesions.^1-6^ The surgery involves both local resection of lesional tissue and deafferentation, leading to complete neuronal isolation of the affected hemisphere. Notably, HT is a more invasive procedure compared to the more commonly studied callosotomy, also known as split-brain surgery. In callosotomy, only the connections between the lesional and contralateral hemispheres are severed to prevent seizure spread or generalization, while each hemisphere remains fully integrated with its afferent inputs and efferent outputs. In contrast, HT effectively eliminates nearly all connections of the affected hemisphere to the outside world.

While ordinary human experience is typically understood as arising from continuous interactions between the brain, the body, and the environment, it remains an open question whether brain activity can support consciousness even when fully isolated from sensory input and motor output—so-called ‘islands of awareness’. Candidate models for studying such islands include ex cranio brains or cerebral organoids, though both remain largely inaccessible for in vivo assessment. In this context, HT represents a clinically available scenario that provides a rare opportunity to investigate whether isolated brain tissue can sustain conscious experience.

Previous work has relied on subjective and objective demarcation criteria to detect consciousness.^7,8^ Subjective criteria typically require introspective self-reporting of mental states, which cannot be achieved in an isolated hemisphere. Instead, investigations of consciousness in isolated hemispheres require objective demarcation criteria based on physically measurable intrinsic mental states, independent of any current bodily or outer experience.

Functional MRI (fMRI) enables the in vivo assessment of brain activity by acquiring blood-oxygen-level-dependent (BOLD) signals over several minutes. While task-based fMRI identifies brain regions engaged during specific tasks, resting-state fMRI (rs-fMRI) characterizes the brain’s intrinsic functional organization at rest, revealing networks of regions that are intrinsically interconnected.^9,10^ These large-scale networks are highly consistent across individuals and are organized along a gradient ranging from unimodal networks supporting specialized functions, such as visual or motor processing, to transmodal networks involved in more complex integrative processes.^11,12^ The most extensively studied and consistently reported network at the transmodal end of this gradient is the default mode network. Activity in the default-mode network increases during internal or introspective mental processing, including remembering, anticipating, and daydreaming.^13-15^ Conversely, individuals with disorders of consciousness exhibit reduced connectivity within the default-mode network, which varies in degree depending on the severity of their clinical condition.^16-19^ The default-mode network is therefore thought to be necessary for consciousness, but a demonstration of default-mode consciousness in the absence of external stimuli is lacking.

In this study, we employ rs-fMRI to examine whether default-mode network activity is sustained in isolated and connected hemispheres following hemispherotomy, and to what extent it reflects the network’s presurgical integrity.

## Materials and methods

### Participants

All individuals treated at the Department of Epileptology of the University Hospital Bonn and who underwent HT at the Department of Neurosurgery between 1992 and 2015 were invited to participate in this study. As a control group, individuals with no history of neurological or psychiatric disorders or prior neurosurgery were recruited. Participants were excluded if head motion during the rs-fMRI scan, quantified as mean framewise displacement, exceeded 0.3 mm. The study was approved by the Medical Ethics Committee of the University Hospital Bonn, and all participants or their legal guardians provided written informed consent. For minors, informed assent was obtained.

### MRI acquisition

All MRI examinations were performed at the Life & Brain Research Centre at the University Hospital Bonn using a 3 Tesla MRI scanner (Magnetom Trio, Siemens Healthineers, Erlangen, Germany) between 2011 and 2015. Due to a scanner update in early 2014, all three scans of one HT individual had to be acquired using a slightly different imaging protocol. For each individual, a structural scan (T1-weighted MPRAGE: magnetization prepared rapid gradient echo) was acquired, parameters before update (all scans except for three scans of one individual): TR = 1570 ms, TE = 3.42 ms, flip angle = 15°, 1 mm isotropic voxel size; parameters after update: TR = 1660 ms, TE = 2.54 ms, flip angle = 9°, 0.8 mm isotropic voxel size and rs-fMRI (echo-planar imaging: EPI, parameters before update: 10 min, 240 images, TR = 2500 ms, TE = 30 ms, flip angle = 90°, 3.0 mm × 3.0 mm × 3.3 mm voxel size; parameters after update (three scans of one individual): 11 min, 300 images, TR = 2200 ms, TE = 30 ms, flip angle = 90°, 3.125 mm × 3.125 mm × 3.565 mm voxel size) were acquired. For rs-fMRI, all participants were instructed to lie still and fixate on a crosshair without thinking about anything or falling asleep. In that case, the scan was aborted, and two further attempts were made.

### Cognitive tests

Cognitive abilities were assessed using the HAWIE-R (Hamburg-Wechsler Intelligenztest)^20^ or HAWIK-III (Hamburg-Wechsler Intelligenztest for Children).^21^ Within these test batteries, full-scale IQ reflects overall general cognitive ability, verbal IQ indexes verbal ability and language-based performance, and performance IQ captures perceptual ability and processing speed.

### MRI preprocessing

Images were converted to *Nifti-1* format and organized according to the *brain imaging data structure* (BIDS) standards.^22^ Lesion masks were manually drawn by a doctor in training who was supervised by an experienced epileptologist to delineate damaged tissue. Lesion masks were initially drawn in axial planes based on the isotropic T1-weighted scans, and were further refined afterwards in coronal and sagittal planes. Image preprocessing was performed using *fMRIPrep* v20.2.0 as detailed in Supplemental Text S1.^23^ Briefly, each T1-weighted image was corrected for intensity non-uniformity, skull-stripped, brain tissue segmented, and spatially normalized to the symmetrical MNI152 standard space through non-linear registration using the MNI152NLin6Sym-template.^24^ Head-motion parameters were estimated, several confounding time-series (framewise displacement, DVARS: temporal derivative of root-mean-square variance over voxels, and three region-wise global signals) were calculated, and blood-oxygen level dependent (BOLD) time-series were finally resampled into standard space. Time-series were cleaned as follows before analyses: detrending, low- (0.08 Hz) and high-pass (0.009 Hz) Butterworth filtering, removal of confounding time-series (translation, rotation, global signal, white matter signal, signal of cerebrospinal fluid), and standardization. A 6 mm full-width at half-maximum Gaussian kernel was applied for spatial smoothing. Prior to the following analyses, images of individuals who had undergone HT were reoriented along the left-right axis such that the isolated hemisphere appeared on the right side, and the connected hemisphere on the left. For the control cohort, all individuals were included both in native orientation and flipped along the left-right axis.

### Group-level independent component analysis

Principles of functional organization based on voxel-wise BOLD time-series were delineated using independent component analysis and seed-based functional connectivity. First, a canonical group independent component analysis, decomposing the voxel-wise BOLD time-series signal in 15 independent components as implemented in *Nilearn*^25^ was carried out in the control cohort. The model order was intentionally set to 15 components to favour stable, large-scale components and prevent overfitting and splitting of the relevant default mode network components. All components were visually assessed, and the component showing the typical default-mode network pattern with activation in the precuneus/posterior cingulate cortex, medial prefrontal cortex, and bilateral angular gyrus was selected. From this component, statistical peak coordinates for the clusters located in the precuneus and the angular gyrus were extracted for both hemispheres.

### Seed-based connectivity analysis and statistical analysis

In the control cohort (including native and left-right flipped scans for all individuals), seed-to-voxel correlation maps were calculated based on the left-hemispheric precuneus seed. In all individuals who had undergone HT, seed-to-voxel correlation maps were calculated based on the left-hemispheric precuneus seed, corresponding to the connected hemisphere after reorientation. For individuals who had undergone hemispherotomy and in whom less than 10% of the voxels from the default-mode network activation in controls overlapped with the individual lesion mask, seed-to-voxel correlation maps were calculated based on the right-hemispheric precuneus seed, corresponding to the isolated hemisphere after reorientation. All seed-to-voxel correlation maps were then *z*-transformed and included in second-level models for group-level inference. Maps with seeds in the connected hemisphere (left after reorientation) and maps with seeds in the isolated hemisphere (right after reorientation) were tested against zero (one-sample *t*-tests), against controls (unpaired, two-sample *t*-tests) and correlated with full-scale IQ scores (one-sample *t*-tests of regression coefficients, if IQ available) and seizure outcome (seizure free versus persisting seizures; unpaired, two-sample *t*-tests). Threshold-free cluster enhancement was performed to correct for family-wise error rate.^26^ To assess robustness, seed-to-voxel correlation maps were additionally computed using alternative seed regions, including the angular gyrus derived from independent component analysis in our control cohort as well as the corresponding Precuneus-4 and Inferior Parietal Lobule-4 regions defined in the Brainnetome atlas.^27^ Spatial overlap between thresholded (*z* > 1.96) seed-to-voxel correlation maps derived from different seeds was quantified using the Dice similarity coefficient.

### Case-level independent component analysis

In both cases, with pre- and postoperative MRI available, independent component analysis decomposing the fMRI signal in 15 independent components was performed on the preoperative scan in native space.^25^ From the visually determined default-mode network component, statistical peak coordinates in each hemisphere were extracted and transformed to both postoperative scans by applying non-linear transforms. In both scans, seed-to-voxel correlation maps using the default-mode network seeds originating from the individual preoperative scans were computed and *z*-transformed.

## Results

### Participants

We included 28 people who underwent HT. Data of two individuals were excluded, as their head movements during the scan exceeded the tolerated limit. Thus, 26 individuals were kept for analysis (median age at surgery 11.5 years, range 0–49; median age at rs-fMRI 20.5 years, range 11–49; 11:15 male:female), including two individuals for whom one preoperative and two postoperative rs-fMRI scans were available. Data from a partly overlapping cohort have previously been published.^28-30^ Table 1 provides a detailed overview of the hemispherotomy cohort. The control group consisted of 24 healthy individuals (median age at rs-fMRI 27.5 years, range 15–58; 12 female).

**Table 1:** Overview over the hemispherotomy cohort

ID	Both hemispheres	Sex	Lesional side	Aetiology	Age at onset (years)	Age at surgery (years)	Age at MRI (years)	Seizure free at MRI	ASM at MRI	Lesion volume (cm^3^)	Full-scale IQ	Verbal IQ	Performance IQ
1*	Yes	M	R	Ganglioglioma	1	26	26	yes	LTG, LCM, PER	302.6	NA	NA	NA
2	No	M	L	Rasmussen’s encephalitis	5	19	21	yes	LTG, OXC	675.4	68	60	80
3	No	M	R	Porencephaly	0	16	19	yes	PHT, LEV, LCM	440.5	47	51	45
4	Yes	F	L	Rasmussen’s encephalitis	6	18	20	yes	None	253.7	82	68	100
5	No	M	L	Porencephaly	9	10	16	yes	None	363.8	72	80	68
6	No	M	R	Porencephaly	0	15	17	yes	LTG	534.6	83	89	79
7	No	F	R	Encephalitis	1	30	45	yes	None	661.4	NA	NA	NA
8	No	F	L	Hemimegalencephaly	0	6	25	yes	LTG	625.3	55	69	54
9	No	F	L	Porencephaly	2	13	21	yes	None	599.4	77	81	72
10	Yes	F	L	Porencephaly	8	18	20	yes	None	177.9	77	90	61
11	No	F	L	Hemimegalencephaly	0	0	18	no	LTG, VPA	784.2	NA	NA	NA
12	No	M	R	Polymicrogyria	9	10	23	yes	None	414.1	94	107	75
13	Yes	F	L	Rasmussen’s encephalitis	3	7	11	yes	None	254.9	78	NA	NA
14	Yes	M	L	Porencephaly	3	33	43	yes	LTG, LEV	358.3	79	90	68
15	No	F	L	Porencephaly	5	9	20	yes	None	377.2	77	81	75
16	No	F	L	Porencephaly	4	11	22	yes	None	344.4	80	84	75
17	No	F	R	Porencephaly	1	10	21	yes	None	658.9	102	110	91
18	No	M	L	Rasmussen’s encephalitis	5	12	18	yes	None	552.1	61	64	67
19	No	M	L	Porencephaly	0	10	18	yes	None	402.6	66	78	58
20	No	F	R	Porencephaly	8	25	36	no	LEV, OXC	652.3	70	81	61
21	Yes	F	R	Hemimegalencephaly	0	0	16	no	LTG	275.4	68	75	66
22	Yes	F	R	Porencephaly	7	16	27	yes	None	255.6	56	67	58
23	No	M	R	Sturge–Weber syndrome	0	1	20	yes	None	449.9	79	80	78
24	No	M	R	Porencephaly	10	29	32	yes	LTG	464.4	59	74	56
25	No	F	L	Porencephaly	0	9	19	yes	None	288.1	83	90	78
26*	Yes	F	L	Porencephaly	0	49	49	yes	LTG, LEV, PRM	283.7	NA	NA	NA

^*^Longitudinal scans available.

### Intact but separated default-mode networks after HT on group level

**Figure 1:**
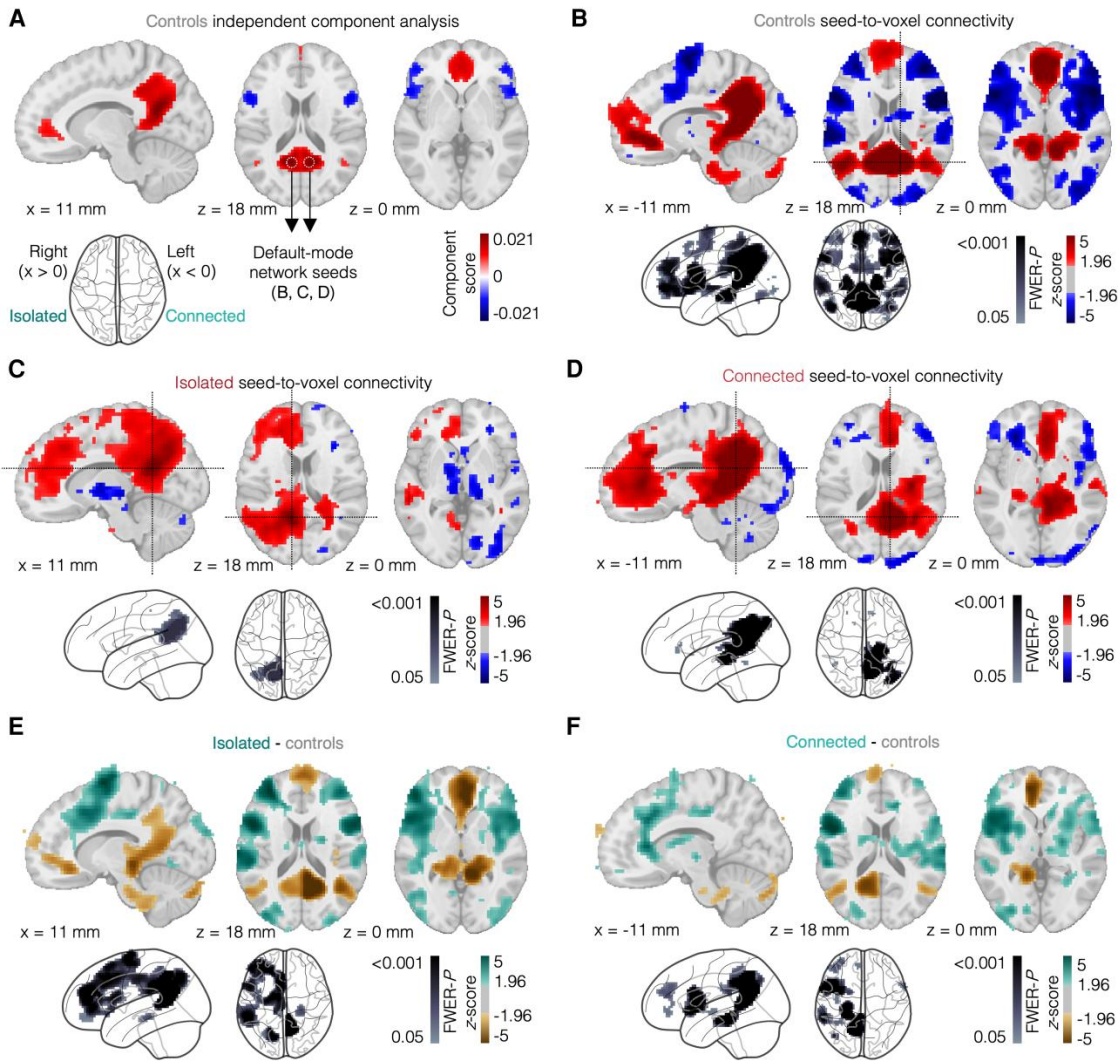
**Seed-to-voxel connectivity analysis.** (**A**) Default-mode network derived from independent component analysis in healthy controls (*N* = 24), subjects included in native and left-right flipped orientation. Peak statistical coordinates located in the posterior cingulate gyrus were (*x*, *y*, *z*) = (11, −56, 18) mm in the right hemisphere and (*x*, *y*, *z*) = (−11, −56, 18) mm in the left hemisphere. Peak statistical coordinates from both hemispheres were used as seeds for seed-to-voxel analyses in controls (**B**, left-hemispheric seed, *N* = 24), isolated (**C**, right hemisphere after reorientation, *N* = 8), and connected (**D**, left hemisphere after reorientation, *N* = 26) hemispheres after HT. A default-mode network pattern was found in connected and isolated hemispheres (one-sample *t*-test). Comparison of seed-to-voxel connectivity between individuals after HT with seed in the isolated hemisphere (**E**) and controls, and between individuals after HT with seed in the connected hemisphere (**F**) and controls (unpaired *t*-test). In (**B–F**), overlays on anatomical images represent uncorrected z-scored statistics and glass-brain inlays with grey colormap depict FWER-corrected *P*-values (*P* < 0.05) after threshold-free cluster enhancement in maximum intensity projection. FWER: family-wise error rate, HT: hemispherotomy.

**Figure 2:**
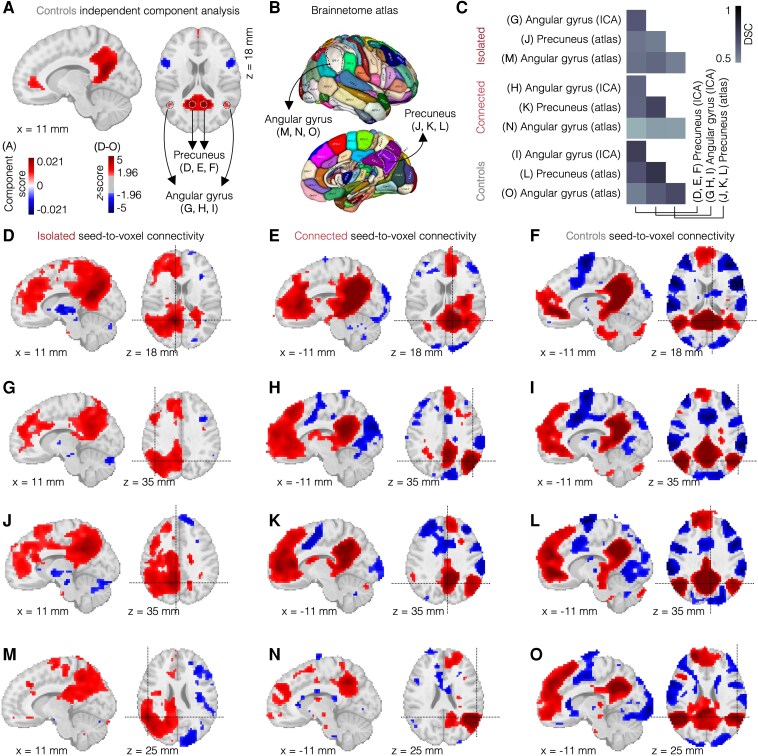
**Seed-to-voxel sensitivity analysis.** (**A**) Seed regions derived from independent component analysis (ICA) in the healthy control cohort (*N* = 24). The primary seed was defined at the statistical peak located in the precuneus, while an alternative seed was identified as a local peak in the angular gyrus. (**B**) Corresponding regions from the Brainnetome atlas were used as atlas-based alternative seeds for sensitivity analyses. A detailed view of the Brainnetome atlas is shown in Supplemental Fig. S2, together with an abbreviation list in Supplemental Table S3. (**C**) Spatial overlap between seed-to-voxel correlation maps, quantified using the Dice similarity coefficient (DSC). (**D–F**) Seed-to-voxel correlation maps derived from the ICA-based precuneus seed. (**G–I**) From the ICA-based angular gyrus seed. (**J–L**) From the atlas-based precuneus seed. (**M–O**) From the atlas-based angular gyrus seed. In all rows, results are shown from left to right for isolated hemispheres (*N* = 8), connected hemispheres (*N* = 26), and healthy controls (*N* = 24). The colourbar labelled **D–O** in panel A applies to all plots in **D–O**. DSC: Dice similarity coefficient; ICA: independent component analysis. Brain plots in (**B**) are reproduced with permission from Oxford University Press from Fan *et al*.^27^

Using independent component analysis, we observed a typical default-mode pattern with activation in the posterior cingulate cortex, medial prefrontal cortex, and angular gyrus in control hemispheres (Fig. 1A). The coordinates of statistical peaks in the posterior cingulate gyrus (*x*, *y*, *z*) = (+/−11 mm, −56 mm, 18 mm) were then used for seed-to-voxel correlation analyses. First, the default-mode network was reproduced in Fig. 1B. In connected hemispheres (all 26 HT cases included), we observed positive correlations between the seed region and medial prefrontal cortex or angular gyrus, but no statistically significant correlations (positive or negative) with the contralateral, isolated hemisphere (Fig. 1D). In isolated hemispheres (8 HT cases included), we observed positive correlations between the seed region and medial prefrontal cortex, but no statistically significant correlations (positive or negative) with the contralateral, connected hemisphere (Fig. 1C). We did not observe any significant differences in seed-to-voxel connectivity in connected hemispheres compared to controls (Fig. 1F). In isolated hemispheres, we did not observe any significant differences in positive seed-to-voxel connectivity between default-mode network regions, but the negative seed-to-voxel connectivity in the isolated hemisphere was significantly weaker in temporal and superior frontal areas than in controls (Fig. 1E). Taken together, these data reveal a pattern of positive correlations within the default-mode network that is identical to controls in both connected and isolated hemispheres. However, negative correlations with regions outside the default-mode network are retained in connected, but not isolated, hemispheres. No statistically significant correlations were observed between IQ scores or seizure outcome and default-mode network connectivity. We observed substantial overlap between default-mode network maps derived from seeding at the statistical peak in the precuneus and from alternative seeds based on independent component analysis in our control sample (angular gyrus), as well as atlas-derived precuneus and angular gyrus seeds (mean Dice coefficients: isolated hemispheres 0.53, connected hemispheres 0.49, controls 0.65; Fig. 2).

### Preoperative individual default-mode networks are split after HT

**Figure 3:**
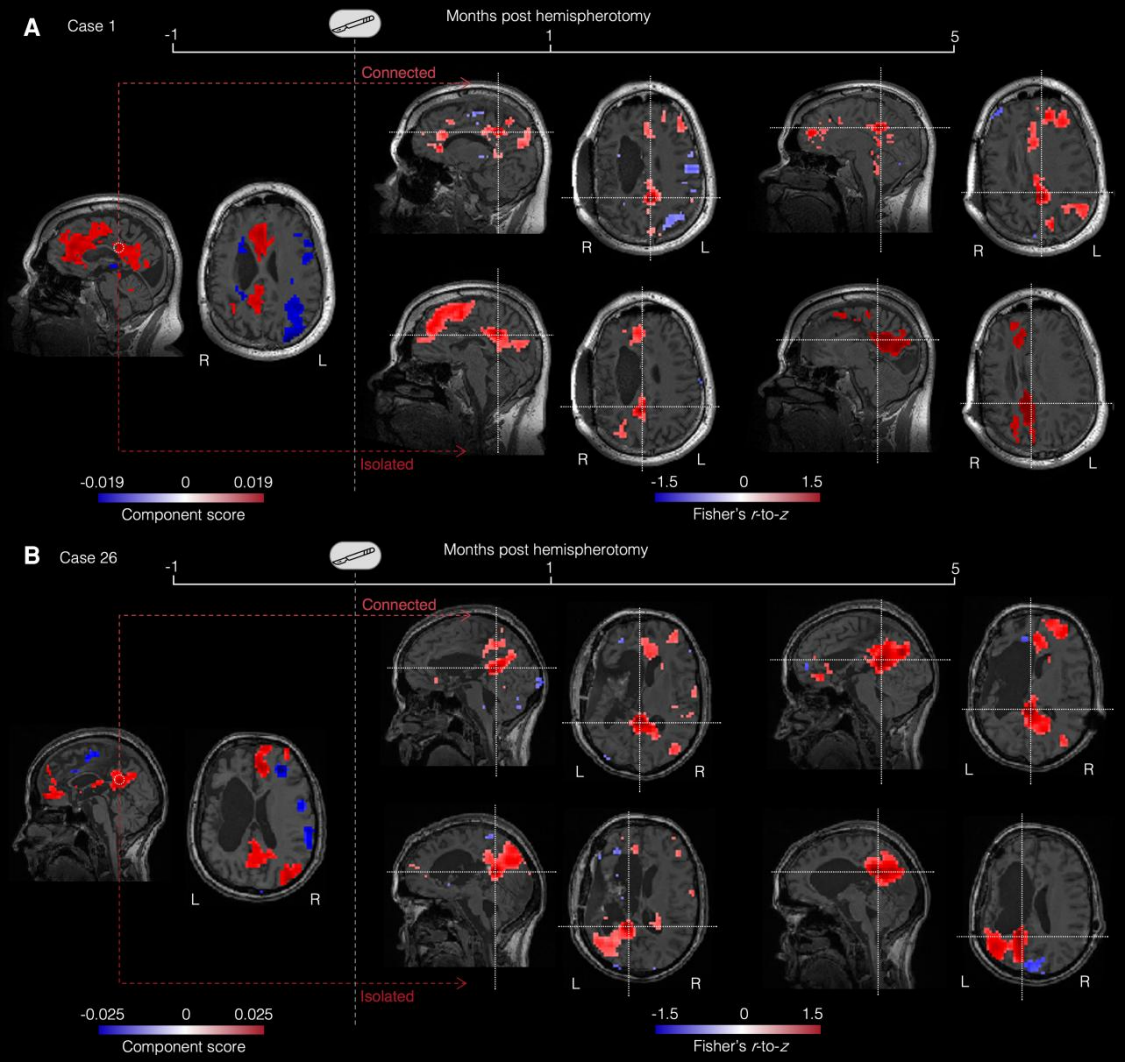
**Pre- and postoperative trajectories for two cases.** Seed-based functional connectivity for 2 cases (**A**: case 1, **B**: case 26) in each hemisphere, 1 and 5 months after hemispherotomy. Seeds for the individual default-mode originate from independent component analysis of the preoperative scan (left). L: left, R: right.

In two cases (cases 1 and 26), we were able to obtain preoperative as well as postoperative data and were therefore able to determine whether postoperative default-mode networks corresponded to preoperative default-mode networks. In both individuals, a typical bilateral default-mode network was identified by independent component analysis following preoperative rs-fMRI. However, activation of the angular gyrus was more prominent in the lesional hemisphere in case 1 (Fig. 3A) and more prominent in the contralesional hemisphere in case 26 (Fig. 3B). When using the statistical peak coordinates of this component as a seed for postoperative analysis, we observed independent default-mode network patterns in each hemisphere in both cases, including at one month and five months post HT. A positive correlation with the medial prefrontal cortex was not seen in the isolated hemisphere in case 26 at five months post HT. Nevertheless, both cases illustrate that the postoperative default-mode networks that are independently observed in both hemispheres correspond to the preoperative default-mode networks.

## Discussion

This report illustrates preserved yet separated default-mode network activity both in the connected and isolated hemispheres following HT, based on group-level inference and individual pre- and post-operative trajectories in two cases.

The state of consciousness of the isolated hemisphere is one of the most intriguing points raised by the discovery of typical default-mode network activity in the isolated hemisphere. Studies investigating functional connectivity in disorders of consciousness, as well as in propofol- and ketamine-induced loss of consciousness, have consistently observed breakdown of functional connectivity within the default-mode network, paired with loss of negative correlations between the default-mode network and other cortical areas as sequelae.^17-19,31,32^ In contrast, we observed an intact default-mode network connectivity pattern with positive correlations between the posterior and anterior cingulate gyri in isolated hemispheres, in alignment with a previous single-case observation.^33^ The absence of negative correlations suggests that, if consciousness is assumed, its level may be reduced compared to the typical awake state.^16-19^

Surprisingly, we did not observe significant associations between default mode network connectivity and cognitive performance, despite prior evidence suggesting a positive relationship between DMN connectivity and cognitive function in healthy adults.^34,35^ This lack of correlation may reflect limited statistical power due to the small sample size. Additionally, it is possible that the functional organization of the DMN after HT differs from that of healthy brains, such that connectivity measures in this population may not relate to cognitive performance in the same way as in neurotypical individuals.

From a methodological perspective, a surface-based approach would have been preferable to the voxel-wise strategy adopted here. However, the substantial anatomical alterations following HT precluded reliable surface reconstruction, rendering such an approach infeasible. A limitation of the voxel-wise approach is that, due to the required spatial smoothing, interhemispheric signal contamination, particularly near the midline, cannot be entirely excluded and should therefore be considered when interpreting our results.

In summary, our findings suggest that a necessary condition for the preservation of consciousness in isolated hemispheres, namely the presence of default-mode network activity, is fulfilled. However, this does not support the reverse inference that such activity alone confirms the presence of consciousness. More direct evidence for isolated consciousness after hemispherotomy could be gained through electrophysiological approaches grounded in integrated information theory, such as assessing the perturbational complexity index in isolated hemispheres.^36,37^

## Supplementary Material

fcag211_Supplementary_Data

## Data Availability

The anonymized data are available from the corresponding author upon reasonable request. Participants did not consent to sharing of their raw MRI data with external investigators. No specific code was generated in this study.
